# Post-16 education and labour market activities, pathways and outcomes (LEO)

**DOI:** 10.23889/ijpds.v8i2.2186

**Published:** 2023-09-14

**Authors:** Oliver Anderson

**Affiliations:** 1 Department for Education, London, United Kingdom

## Objectives

This is descriptive analysis of post-16 education and labour market activities, pathways and outcomes based on different socioeconomic, demographic and education factors. This research uses powerful administrative data from England to carry out analysis of over 3.6 million individuals doing their General Certificate of Secondary Education exams (GCSEs) between 2002 and 2007.

## Method

Education records and combined with tax and social security records (from the National Pupil Database). Longitudinal Education Outcomes (LEO) data allowing these individuals to be tracked in their post compulsory education over a 10-15 year period. The analysis makes comparisons using of a range of background characteristics including socioeconomic status, special educational needs (SEN) status, gender, ethnicity, first language and location (region). It also observes how these differ for different education levels, doing comparisons of: 1) graduates and non-graduates and 2) non-graduates achieving level 3 or above and level 2 or below.

## Results

It finds that Post 16 (i.e. compulsory in England) Education and labour market pathways are incredibly diverse and they differ significantly based on individual characteristics. Higher levels of education lead to better labour market outcomes and most sub-groups achieving a higher education level leads to better labour market outcomes than their comparators (with different characteristics). For example, there are higher proportions of graduates that were Free School Meals eligible (a proxy for lower socioeconomic status) in employment and lower proportions claiming benefits than non-Free School Meals (FSM) eligible non-graduates, 15 years after their GCSEs (63 percent versus 58 percent and five versus nine percent respectively). Of those in employment, the FSM eligible graduates earn around £5,000 more per year than non-FSM eligible non-graduates and their earnings potential seem to have different trajectories.

## Conclusion

There are some interesting and insightful findings, but it should be noted that findings are descriptive. It is recommended that in depth, technical analysis is undertaken to explored further.

